# Determination of internal controls for quantitative gene expression of *Spodoptera litura* under microbial pesticide stress

**DOI:** 10.1038/s41598-024-56724-9

**Published:** 2024-03-13

**Authors:** Shuang Wu, Yunmi Luo, Zhihong Zeng, Ying Yu, Shicai Zhang, Yan Hu, Lei Chen

**Affiliations:** Institute of Vegetable and Flower Research, Chongqing Academy of Agricultural Sciences, Chongqing, 401329 China

**Keywords:** PCR-based techniques, Gene expression analysis, Entomology

## Abstract

Quantitative real-time polymerase chain reaction (qRT-PCR) has become a commonly used method for the quantification of gene expression. However, accurate qRT-PCR analysis requires a valid internal reference for data normalization. To determine the valid reference characterized with low expression variability among *Spodoptera litura* samples after microbial pesticide treatments, nine housekeeping genes, glyceraldehyde-3-phosphate dehydrogenase (*GAPDH*), arginine kinase, ubiquitin C, actin-5C (*ACT5C*), actin, ribosomal protein S13 (*RPS13*), tubulin, acidic ribosomal protein P0 (*RPLP0*) and ubiquinol-cytochrome c reductase, were evaluated for their suitability using geNorm, Normfinder, BestKeeper, RefFinder and the comparative delta CT methods in this study. *S. litura* larvae after direct treatment (larvae were immersed in biopesticides), indirect treatment (larvae were fed with biopesticide immersed artificial diets) and comprehensive treatment (larvae were treated with the first two treatments in sequence), respectively with *Metarhizium anisopliae*, *Empedobacter brevis* and *Bacillus thuringiensis*, were investigated. The results indicated that the best sets of internal references were as follows: *RPLP0* and *ACT5C* for direct treatment conditions; *RPLP0* and *RPS13* for indirect treatment conditions; *RPS13* and *GAPDH* for comprehensive treatment conditions; *RPS13* and *RPLP0* for all the samples. These results provide valuable bases for further genetic researches in *S. litura*.

## Introduction

*Spodoptera litura* Fabricius (Lepidoptera: Noctuidae) is a globally distributed polyphagous pest that damages approximately 389 species of plants including vegetables, fruits, cotton, and tobacco; the most commonly affected plants are crop species^1^. The high reproductive potential of this species and intense nutritional requirements of its larva means that most damage is incurred over a short period of time^2–6^. Outbreaks of *S. litura* have been reported in many countries, including China, India, Pakistan, Japan, Indonesia and Australia^7–13^. Over the past few decades, chemical control has been utilized as the main strategy for managing *S. litura*. However, the development of resistance to chemical pesticides in this species leads to subsequent management failure, posing a serious threat to global agricultural production^14–17^.

Over recent years, more biological approaches have been developed to effectively control *S. litura*^18,19^. Biopesticides are the biological agents that are used to control pests, and are derived from fungi, bacteria, viruses, plants, animals, and certain minerals. Of all biopesticides, microbial pesticides are becoming increasingly more important and have gained significant popularity because they are safe and environmentally friendly^20,21^. The most widely used microbial pesticides are strains of *Bacillus*, *Metarhizium* and *Empedobacter*, these have all been demonstrated to be effective against various Lepidoptera pests^22–28^. However, in a manner similar to that of chemical control methods, some target herbivores, including *S. litura*, have developed resistance to microbial pesticides, including *B. thuringiensis*^29–33^.

Molecular technologies, especially quantitative real-time polymerase chain reaction (qRT-PCR), have been used extensively in genetic studies relating to the mechanisms of immunity in insects^34–36^. Significant changes in the levels of gene expression can reflect biological changes in insects under different experimental conditions. To investigate the specific changes in immune-associated genes in *S. litura* under different microbial pesticide stress conditions, especially under different exposure treatments to pesticide, valid internal references for qRT-PCR analyses were screened. One group of *S. litura* larvae was treated directly with *M. anisopliae*, *E. brevis* and *B. thuringiensis*, respectively, by immersion in biopesticide; another group of larvae was indirectly treated by feeding the larvae with artificial diets immersed in biopesticide; the final group of larvae was treated using the first two treatment modes in sequence; we referred to this strategy as the comprehensive treatment. Nine housekeeping genes, glyceraldehyde-3-phosphate dehydrogenase (*GAPDH*), arginine kinase (*AK*), ubiquitin C (*UBC*), actin-5C (*ACT5C*), actin (*ACT*), ribosomal protein S13 (*RPS13*), tubulin (*TUB*), acidic ribosomal protein P0 (*RPLP0*) and ubiquinol-cytochrome c reductase (*UCCR*), in samples of *S. litura* following different treatments with microbial pesticide, were evaluated for their suitability as normalization references using geNorm, Normfinder, BestKeeper, RefFinder and the comparative delta CT methods.

## Results

### Qualities of total RNA

Total RNAs were extracted from *S. litura* after 6, 12, 24, 48 and 72 h of direct treatments, and after 24, 48, 72 h of indirect treatments and comprehensive treatments, respectively. The concentrations and purities of the total RNA isolated from *S. litura* samples were determined with a GeneQuant Pro RNA/DNA Calculator (GE Healthcare, Piscataway, NJ, USA). The total RNA concentrations ranged from 711.3 to 1654.8 ng·μL^−1^ for the directly treated samples, from 810.9 to 1674.3 ng·μL^−1^ for the indirectly treated samples, and from 767.9 to 1284.1 ng·μL^−1^ for the comprehensive groups. The A260/A280 ratios ranged from 2.04 to 2.11 for the directly treated samples, from 1.91 to 2.11 for the indirectly treated samples, and from 1.93 to 2.12 for the comprehensive groups (Supplementary Table S1). The integrity of all total RNA samples was confirmed by 1.0% agarose gel electrophoresis.

### PCR amplification efficiencies

For each primer pair, the single peak melting curves indicated that a unique product was amplified (Supplementary Fig. S1). The products were sequenced and BLAST searches were performed at http://www.ncbi.nlm.nih.gov/blast/. BLASTn revealed that the products had 100% identity with the fragment sequences on which the primer design as based. The PCR amplification efficiency and the coefficient of determination (*R*^2^) were 99.40 and 0.9971 for *GAPDH*, 109.39 and 0.9972 for *AK*, 109.53 and 0.9998 for *UBC*, 94.00 and 0.9994 for *ACT5C*, 96.86 and 0.9984 for *ACT*, 106.41 and 0.9977 for *RPS13*, 91.22 and 0.9985 for *TUB*, 102.18 and 0.9968 for *RPLP0*, 106.64 and 0.9998 for *UCCR*, respectively (Supplementary Fig. S2).

### Expression profiles of the candidate reference genes

**Figure 1:**
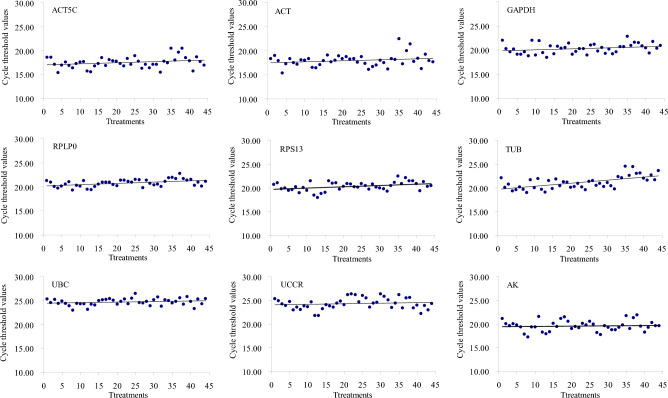
Regression lines for the nine candidate reference genes. Each dot indicates the mean of duplicate samples (n = 3). The most stable reference gene has the closest fit to the regression line.

**Figure 2:**
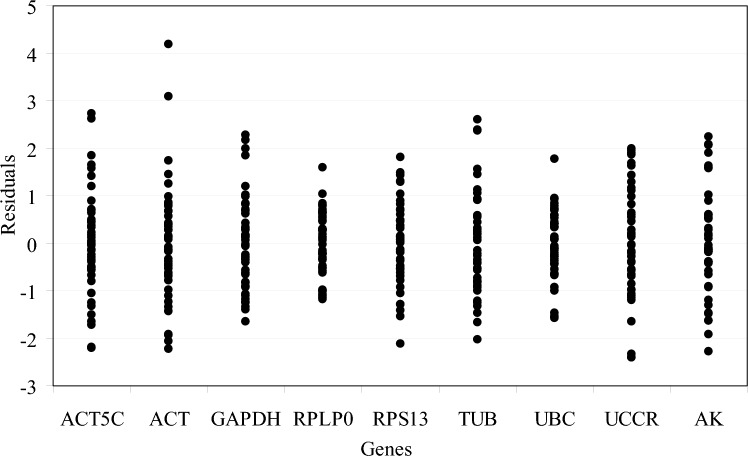
Scatterplot of residuals analysis. The residuals of CT values were evaluated by linear regression in Fig. 1 and the difference between the actual value and the calculated value for each gene.

According to the results of crude expression levels and stability of each gene from our previous research on *S. litura* transcriptome under microbial pesticide stress, the nine housekeeping genes (*GAPDH*, *AK*, *UBC*, *ACT5C*, *ACT*, *RPS13*, *TUB*, *RPLP0* and *UCCR*) were chosen to serve as the candidate reference genes for this study. The expression levels of the nine candidate reference genes in *S. litura* samples were investigated using a SYBR Green-based qPCR assay which was performed in triplicate. The entire experiment was then repeated. The mean cycle threshold (CT) values ranged from 15.35 (*ACT*) to 26.50 (*UBC*) in all samples, from 15.37 to 20.48 for *ACT5C*, from 15.35 to 22.41 for *ACT*, from 18.43 to 22.83 for *GAPDH*, from 19.30 to 22.76 for *RPLP0*, from 17.92 to 22.46 for *RPS13*, from 18.96 to 24.56 for *TUB*, from 22.99 to 26.50 for *UBC*, from 21.80 to 26.38 for *UCCR*, and from 17.23 to 21.95 for *AK* (Fig. 1). The CT values in highest to lowest order were as follows: *UBC*, *UCCR*, *TUB*, *RPLP0*, *RPS13*, *GAPDH*, *AK*, *ACT* and *ACT5C*. The residuals of CT values were evaluated by linear regression and the difference between the actual value and the calculated value for each gene (Fig. 1); CT values were ranked as follows (highest to lowest stability): *RPLP0*, *UBC*, *GAPDH*, *RPS13*, *UCCR*, *AK*, *TUB*, *ACT5C* and *ACT*, according to the distributions of residuals (Fig. 2).

### Analysis of gene expression stability

#### Direct treatment

**Figure 3:**
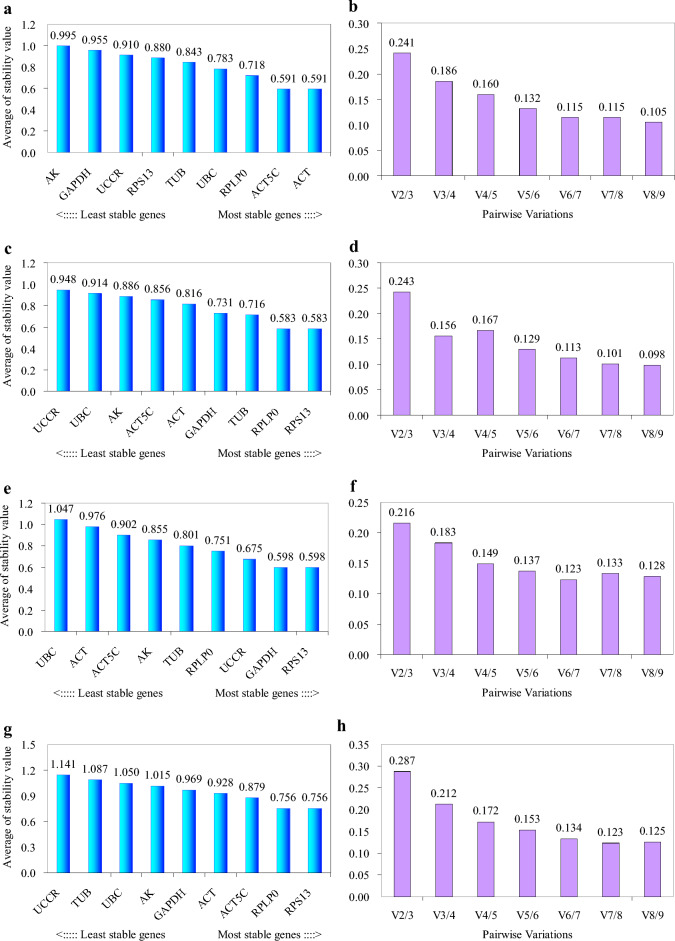
The average expression stability value M and the pairwise variation V of candidate genes as determined by geNorm analysis. (**a** and **b**) direct treatment conditions; (**c** and **d**) indirect treatment conditions; (**e** and **f**) comprehensive treatment conditions; (**g** and **h**) all treatments.

**Figure 4:**
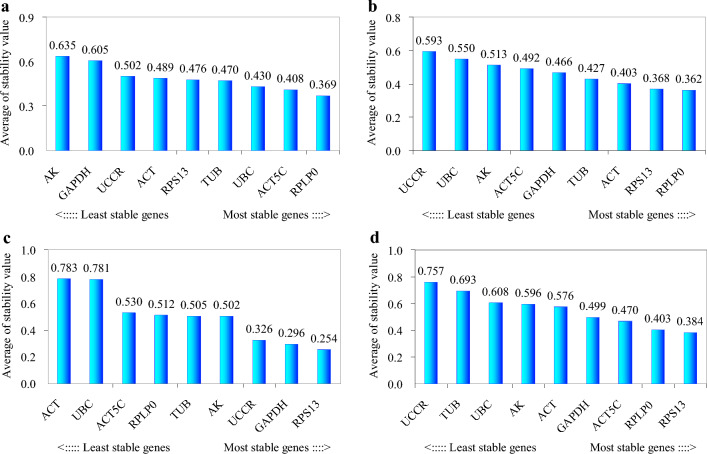
Expression stabilities of the candidate reference genes as determined by Normfinder software. **a** direct treatment conditions; **b** indirect treatment conditions; **c** comprehensive treatment conditions; **d** all treatments.

**Figure 5:**
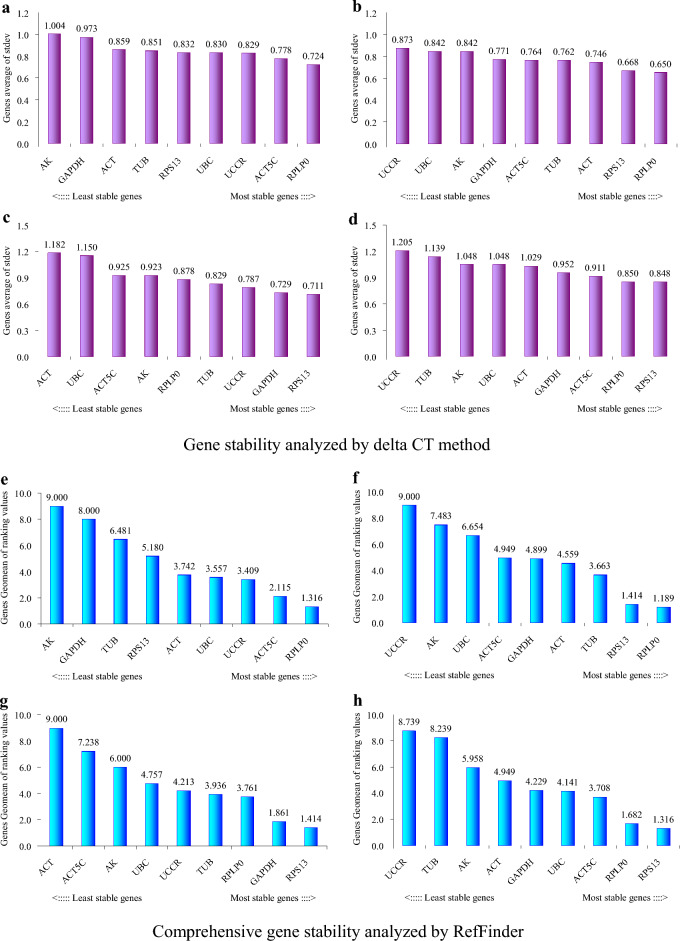
Expression stabilities of the candidate reference genes as determined by the comparative delta CT method (**a**–**d**) and RefFinder software (**e**–**h**). (**a** and **e**) direct treatment conditions; (**b** and **f**) indirect treatment conditions; (**c** and **g**) comprehensive treatment conditions; (**d** and **h**) all treatments.

According to geNorm analysis, the stability rankings from the most stable to the least stable gene were *ACT*, *ACT5C*, *RPLP0*, *UBC*, *TUB*, *RPS13*, *UCCR*, *GAPDH* and *AK* (Fig. 3a). Furthermore, geNorm analysis revealed that the pair-wise variation value V5/6 was below the proposed 0.15 cut-off (Fig. 3b). This result suggested that the average of the top five genes would be the optimal normalization factor for further experiments. Normfinder analysis identified *RPLP0*, *ACT5C* and *UBC* as the most stable genes (Fig. 4a). According to the standard deviation (SD) and coefficient of variation (CV) values in Table 1, BestKeeper analysis identified *RPLP0*, *UBC* and *UCCR* as the most stable genes. The stability rankings generated by the delta CT and RefFinder methods identified *RPLP0*, *ACT5C* and *UCCR* as the most stable genes (Fig. 5a,e). Moreover, all software tools identified *AK* as the least stable gene while most software tools identified *RPLP0* and *ACT5C* as the top two most stable genes.

**Table 1:** Stability of candidate reference genes as determined by BestKeeper software.

Gene	Direct treatment conditions				Indirect treatment conditions				Comprehensive treatment conditions				All samples			
	Rank	SD	CV	*r*	Rank	SD	CV	*r*	Rank	SD	CV	*r*	Rank	SD	CV	*r*
*ACT5C*	5	0.74	4.32	0.868	6	0.63	3.69	0.898	8	1.08	5.94	0.946	7	0.88	5.02	0.910
*ACT*	4	0.71	3.99	0.772	8	0.72	4.11	0.875	9	1.35	7.22	0.934	6	0.86	4.79	0.857
*GAPDH*	8	0.94	4.69	0.758	4	0.58	2.90	0.658	3	0.62	2.96	0.935	4	0.83	4.11	0.813
*RPLP0*	1	0.51	2.51	0.874	2	0.50	2.38	0.812	2	0.54	2.53	0.743	2	0.62	2.99	0.842
*RPS13*	6	0.80	4.01	0.846	1	0.42	2.09	0.740	4	0.64	3.03	0.942	3	0.75	3.72	0.883
*TUB*	7	0.86	4.20	0.828	3	0.52	2.51	0.604	5	0.78	3.43	0.851	9	1.12	5.28	0.810
*UBC*	2	0.53	2.15	0.716	5	0.61	2.44	0.627	1	0.53	2.12	0.317	1	0.57	2.32	0.550
*UCCR*	3	0.70	2.96	0.816	9	0.79	3.10	0.716	7	0.91	3.76	0.945	8	0.95	3.91	0.617
*AK*	9	0.98	4.98	0.809	7	0.64	3.31	0.690	6	0.90	4.51	0.844	5	0.84	4.30	0.766

### Indirect treatment

According to geNorm analysis, the stability rankings from the most stable to the least stable gene were *RPS13*, *RPLP0*, *TUB*, *GAPDH*, *ACT*, *ACT5C*, *AK*, *UBC* and *UCCR* (Fig. 3c). Furthermore, geNorm analysis revealed that the pair-wise variation value V5/6 was below the proposed 0.15 cut-off (Fig. 3d). This result suggested that the average of the top five genes would be the optimal normalization factor for further experiments. Normfinder and delta CT methods identified *RPLP0*, *RPS13* and *ACT* as the most stable genes (Figs. 4b, 5b). According to the standard deviation and CV values in Table 1, BestKeeper software identified *RPS13*, *RPLP0* and *TUB* as the most stable genes; these were the same as those identified by geNorm. RefFinder software identified *RPLP0*, *RPS13* and *TUB* as the top three most stable genes (Fig. 5f); these results were similar to those derived from geNorm and BestKeeper. Moreover, all of the software tools identified *UCCR* as the least stable gene, and identified *RPLP0* and *RPS13* as the top two most stable genes.

### Comprehensive treatment

According to geNorm analysis, the stability rankings from the most stable to the least stable gene were *RPS13*, *GAPDH*, *UCCR*, *RPLP0*, *TUB*, *AK*, *ACT5C*, *ACT* and *UBC* (Fig. 3e). Furthermore, geNorm analysis revealed that the pair-wise variation value V4/5 was below the proposed 0.15 cut-off (Fig. 3f). This result suggested that the average of the top four genes would be the optimal normalization factor for further experiments. Normfinder and delta CT methods also identified *RPS13*, *GAPDH* and *UCCR* as the top three most stable genes (Figs. 4c, 5c); these results were the same as those generated by geNorm. According to the standard deviation and CV values in Table 1, BestKeeper software identified *UBC*, *RPLP0* and *GAPDH* as the most stable genes. It’s notable that *UBC* was identified as the least stable gene by geNorm. RefFinder analysis identified *RPS13*, *GAPDH* and *RPLP0* as the most stable genes (Fig. 5g). Moreover, all software tools, except for geNorm, identified *ACT* as the least stable gene. Most of the programs identified *RPS13* and *GAPDH* as the top two most stable genes.

### All treatments

Next, we investigated the stability rankings of the nine candidate reference genes for all treated *S. litura* samples. According to geNorm analysis, the stability rankings from the most stable to the least stable gene were *RPS13*, *RPLP0*, *ACT5C*, *ACT*, *GAPDH*, *AK*, *UBC*, *TUB* and *UCCR* (Fig. 3g). In addition, geNorm analysis revealed that the pair-wise variation value V6/7 was below the proposed 0.15 cut-off (Fig. 3h). This result indicated that the average of the top six genes would be the optimal normalization factor for further experiments. All software packages, except for BestKeeper, identified *RPS13*, *RPLP0* and *ACT5C* as the most stable genes (Figs. 4d, 5d,h). *UBC*, *RPLP0* and *RPS13* were the most stable genes generated by the BestKeeper software (Table 1). Moreover, all software packages, except for BestKeeper, identified *UCCR* as the least stable gene.

### Validation of the candidate internal reference genes

**Figure 6:**
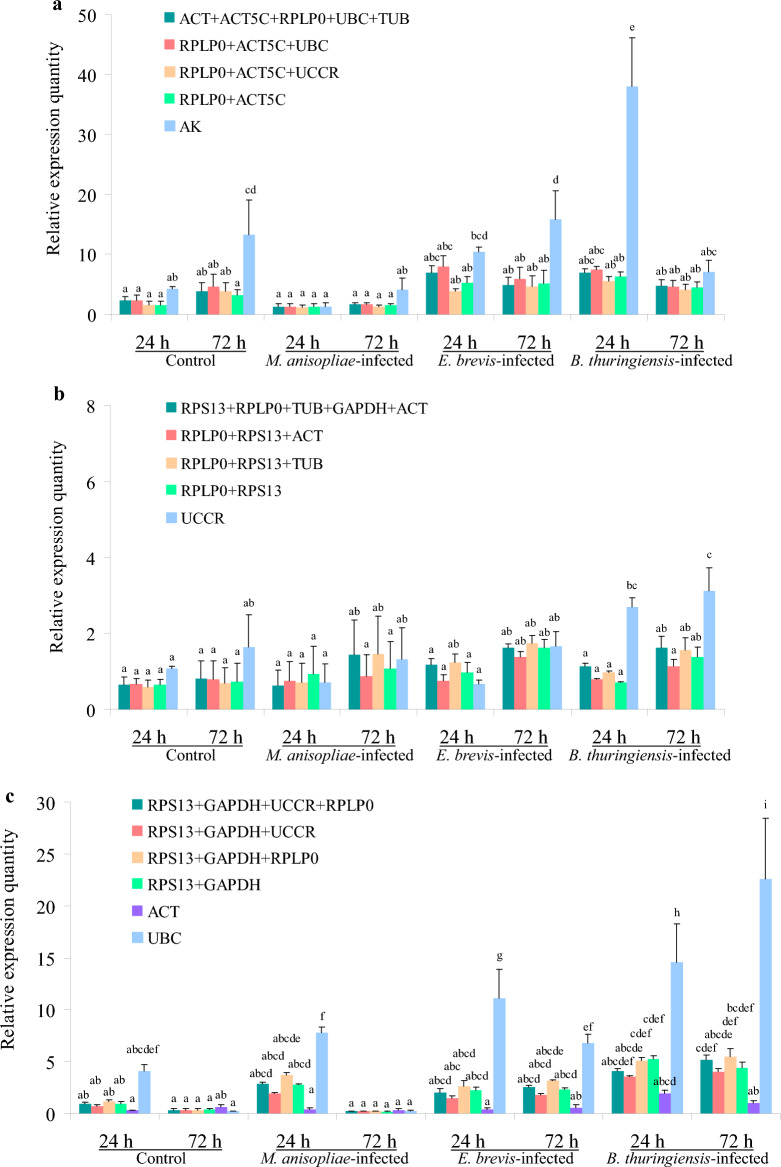

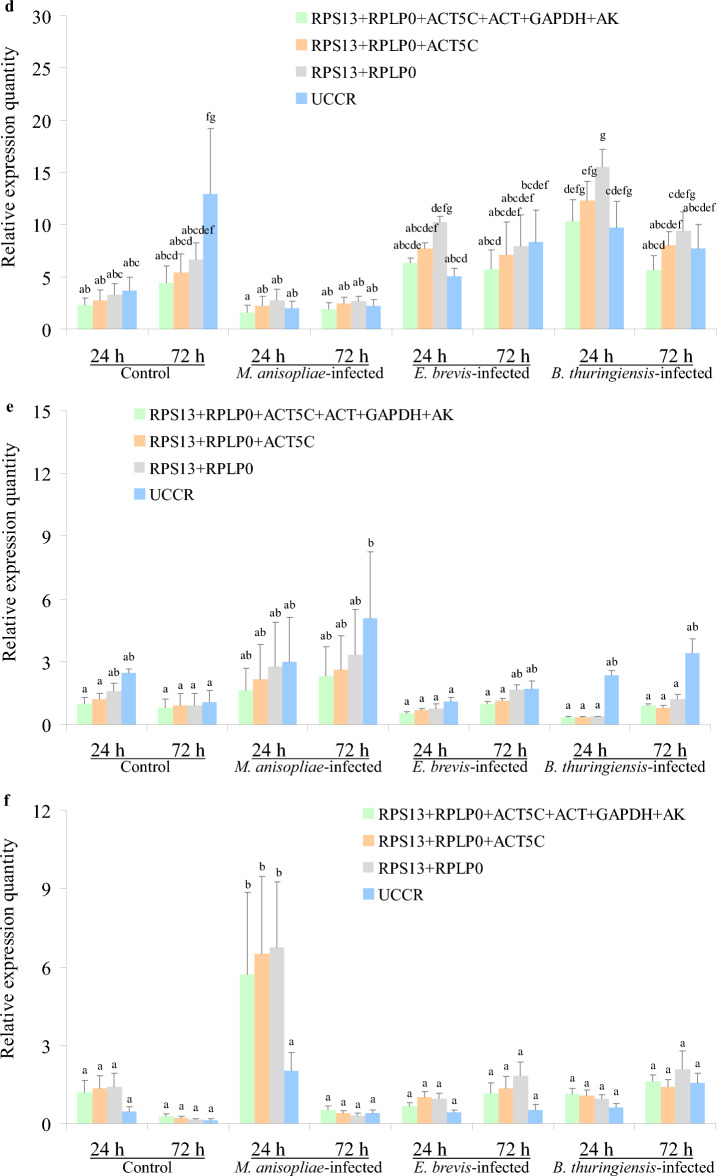
The relative expression levels of C-Type Lectin genes in *S. litura* after 24 h and 72 h of treatment with microbial pesticide. (**a** and **d**) direct treatment conditions; (**b** and **e**) indirect treatment conditions; (**c** and **f**) comprehensive treatment conditions; (**a**–**c**) *SlCTL* expression levels when normalized by the internal references determined from different treatment conditions; (**d**-**f**) *SlCTL* expression levels when normalized by the internal references determined from all treatments. Values are expressed as mean ± SE (n = 3). Different letters above each bar indicate statistical differences, as determined by ANOVA followed by Duncan’s multiple range test (*P* < 0.05).

Previous research has shown that C-type lectins (*CTLs*) participate in pathogen recognition in insects and play diverse roles in a range of immune responses, including opsonization, agglutination, nodule formation, encapsulation, phagocytosis, melanization, prophenoloxidase activation and homeostatic maintenance of the gut microbiome^36–38^. In addition, the expression levels of *CTLs* in *S. litura* were demonstrated to undergo change following fungal or bacterial infections^36^. In the present study, we evaluated the relative expression levels of *SlCTL* in *S. litura* after 24 h and 72 h of microbial pesticide treatments, using the multiple genes recommended by geNorm, the top three and the top two most stable genes, and the least stable gene identified by most of software packages, as internal references for data normalization. Figure 6 shows that there was no significant difference among the relative expression levels of *SlCTL* normalized by the following combinations: *ACT* + *ACT5C* + *RPLP0* + *UBC* + *TUB*, *RPLP0* + *ACT5C* + *UBC*, *RPLP0* + *ACT5C* + *UCCR* and *RPLP0* + *ACT5C* for the direct treatment (*P* > 0.05) (Fig. 6a); *RPS13* + *RPLP0* + *TUB* + *GAPDH* + *ACT*, *RPLP0* + *RPS13* + *ACT*, *RPLP0* + *RPS13* + *TUB* and *RPLP0* + *RPS13* for the indirect treatment (*P* > 0.05) (Fig. 6b) ; *RPS13* + *GAPDH* + *UCCR* + *RPLP0*, *RPS13* + *GAPDH* + *UCCR*, *RPS13* + *GAPDH* + *RPLP0* and *RPS13* + *GAPDH* for the comprehensive treatment (*P* > 0.05) (Fig. 6c). Moreover, the expression levels of *SlCTL* when normalized by the least stable genes (*AK*, *UCCR*, *ACT* and *UBC*) under different treatments were significantly different from the levels normalized by the other gene combinations (*P* < 0.05) (Fig. 6a–c). When take all the treatment samples into consideration, the gene combinations, *RPS13* + *RPLP0* + *ACT5C* + *ACT* + *GAPDH* + *AK*, *RPS13* + *RPLP0* + *ACT5C* and *RPS13* + *RPLP0*, as well as the least stable gene *UCCR*, were used for normalizing the expression levels of *SlCTL*. Figure 6d,f show that the relative expression levels of *SlCTL* normalized by *UCCR* were significantly different from the levels normalized by the other gene combinations (*P* < 0.05). These results demonstrated that using the top two most stable genes was valid for the normalization of *SlCTL* expression levels under the experimental conditions used herein, and that the use of inappropriate internal references would lead to inaccurate experimental results.

## Discussion

Housekeeping genes, such as 18S ribosomal RNA (*18S rRNA*), elongation factor 1 alpha (*EF1-a*), ribosomal protein L18 (*RPL18*), ribosomal protein S18 (*RPS18*), beta actin (*β-actin*), glyceraldehyde-3-phosphate dehydrogenase (*GAPDH*), alpha tubulin (*α*-*Tub*), beta tubulin (*β*-*Tub*), TATA-Box binding protein (*TBP*), and glucose-6-phosphate dehydrogenase (*G6PDH*), are commonly used as internal reference genes in insect studies. Of these, *ACT*, RPS18 and *GAPDH* have previously been identified as the most stable genes in *Apis mellifera* following infection with *Escherichia coli*^39^. *RPS3*, *RPS18*, and *RPL13a* have been identified as the most stable genes in *Tribolium castaneum* following infection with *Beauveria bassiana*^40^. For gene studies involving *S. litura*, the *β*-actin gene was previously used as an internal reference to determine target gene expression patterns under zinc and *Nomuraea rileyi* stress^34,41^. EF-1 has been used to normalize expression levels following infection with *N. rileyi*, *SpltNPV* and *B. thuringiensis*, respectively^36^, and other reference genes were identified across different biotic and abiotic experimental conditions^35,42^.

Previous studies on *S. litura* indicated that some commonly used housekeeping genes exhibited significant variation in expression under different experimental conditions^35,42^. To investigate the molecular mechanisms of immunity in *S. litura* under different microbial pesticide stress conditions, especially in terms of different treatment modes of exposure to pesticide, we validated a range of internal reference genes for data normalization in the present study. Based on our previous research on the *S. litura* transcriptome under microbial pesticide stress, the nine housekeeping genes, *GAPDH*, *AK*, *UBC*, *ACT5C*, *ACT*, *RPS13*, *TUB*, *RPLP0* and *UCCR*, with appropriate FPKM values (fragments per kilo base of transcript per million fragments mapped), were selected to serve as candidate reference genes. Since an appropriate expression level (a CT value between 15 and 30) is important for analyzing internal reference genes^43–47^, the CT values of the nine candidate reference genes were confirmed in all of the treated *S. litura* samples, and the results showed that the average CT values ranged from 15.35 (*ACT*) to 26.50 (*UBC*) (Fig. 1).

The stabilities of the nine candidate reference genes were analyzed by geNorm, Normfinder, BestKeeper, RefFinder and the comparative delta CT methods. The results indicated that in most cases the top three most stable genes ranked by BestKeeper under each treatment condition were different from those obtained from other software packages. The relative expression levels of *SlCTL*, when normalized by *UBC* under comprehensive treatment conditions, were significantly different from the levels normalized by the other references (*P* < 0.05) (Fig. 6c), in particular, *UBC* was identified as the most stable gene by BestKeeper software (Table 1), but was identified as the least stable gene when ranked by geNorm (Fig. 3e). Consequently, the internal reference genes recommended by BestKeeper should not be applied for further analyses under the experimental conditions described herein. Furthermore, the geNorm manual states that the application of the three best reference genes is a valid normalization strategy in most cases. Therefore, the relative expression levels of *SlCTL* were also normalized by the combination of the top three most stable genes under each treatment condition. Figure 6 showed that all of the detected gene combinations were valid for the normalization of *SlCTL* expression levels under the experimental conditions used herein; there was no significant difference between the *SlCTL* expression levels normalized by the combinations of the top three and the top two most stable genes (*P* > 0.05). Finally, the preferable reference genes across different treatment conditions according to our overall analysis were as follows: *RPLP0* and *ACT5C* for direct treatment conditions; *RPLP0* and *RPS13* for indirect treatment conditions; *RPS13* and *GAPDH* for comprehensive treatment conditions; along with *RPS13* and *RPLP0* for all samples.

*RPLP0* is located in the 60S ribosomal subunit, which plays a role in the association of elongation factors with the ribosome during protein synthesis^48–50^, DNA repair^51^, gene expression regulation^52^ and O_2_ consumption cycles^53^. *RPS13* is located in the 40S ribosomal subunit and plays a role in peptide chain elongation and translocation of the mRNA:tRNA complex^54^. These two ribosomal proteins were alternately ranked as the most stable gene in *S. litura* under the experimental conditions used herein. As with studies performed on other insect species, ribosomal protein genes have always been validated as internal controls for qRT-PCR^39,42,55–61^.

In summary, the internal controls applied for qRT-PCR studies on *S. litura* under different microbial pesticide stress conditions, especially with different treatment modes of exposure to pesticide, were different. The selection of a valid normalization gene is particularly important for experimental reliability. Our findings provide valuable bases for further research on genes in *S. litura*.

## Materials and methods

### Insects and biopesticides

*S. litura* were collected as larvae from cabbage fields at the National Center for Vegetable Improvement (Chongqing, China) in October 2022. The larvae were identified as *S. litura* by analyzing larval morphology, especially according to the two subtriangular dark spots on each segment of the larva, except for the prothorax. Ten generations of *S. litura* were reared in the laboratory to minimize the effects of field environments. Larvae were reared on a soybean and wheat bran-based artificial diet^62^. Insects were kept at 26 °C, with a 12 h photoperiod, and a relative humidity of 70%. Three biological pesticide products, 8.0 × 10^9^ spores·mL^-1^ of *Metarhizium anisopliae* CQMa421 OD (Chongqing Julixin Bioengineering Co., Ltd, Chongqing, China), 1.0 × 10^10^ spores·mL^-1^ of *Empedobacter brevis* GXW15-4 SC (Zhenjiang Runyu Biological Science and Technology Development Co., Ltd, Jiangsu, China), and 16,000 IU·mg^-1^ of *Bacillus thuringiensis* WP (King Biotec Corp. Hubei, China), were used in this study. Since our preliminary experiments showed that 50% of *S. litura* larvae could survive for 7 days after immersion treatment with 2.00 × 10^7^ spores·mL^-1^ of *M. anisopliae*, 6.25 × 10^7^ spores·mL^-1^ of *E. brevis* and 2.50 mg·mL^-1^ of *B. thuringiensis*, respectively, these concentrations of biopesticides were used for subsequent experiments to ensure sufficient time and larvae before they pupated.

### Biopesticide stress

#### Direct treatment

Ten fourth instar day 1 larvae were immersed in *M. anisopliae* (2.00 × 10^7^ spores·mL^-1^), *E. brevis* (6.25 × 10^7^ spores·mL^-1^) and *B. thuringiensis* (2.50 mg·mL^-1^), respectively, for 10 s and allowed to air dry at room temperature. The larvae immersed in distilled water were used as control. Three replicates were prepared for each sample. The larvae were collected at 6, 12, 24, 48 and 72 h post-infection.

### Indirect treatment

Ten fourth instar day 1 larvae were fed with 1.0 cm^3^ of an artificial diet immersed in *M. anisopliae* (2.00 × 10^7^ spores·mL^-1^), *E. brevis* (6.25 × 10^7^ spores·mL^-1^) and *B. thuringiensis* (2.50 mg·mL^-1^), respectively, and the larvae fed with distilled water immersed artificial diets were used as control. Untreated artificial diets were provided when the immersed diets were exhausted. Three replicates were prepared for each sample. Since it would take around 24 h for the larvae to finish the biopesticides-immersed artificial diets, the larvae after 24, 48 and 72 h treatment were collected, respectively.

### Comprehensive treatment

Ten fourth instar day 1 larvae were immersed in *M. anisopliae*, *E. brevis* and *B. thuringiensis*, and fed with 1.0 cm^3^ of an artificial diet immersed in biopesticide, respectively. The larvae and diets treated with distilled water were used as control. Untreated artificial diets were provided when the immersed diets were exhausted. Three replicates were prepared for each sample. Since it would take around 24 h for the larvae to finish the biopesticides-immersed artificial diets, the larvae after 24, 48 and 72 h treatment were collected, respectively.

### Total RNA extraction and cDNA synthesis

Total RNA was isolated from five larvae with TRIzol reagent (Invitrogen, Carlsbad, CA, USA). First-strand cDNAs were then synthesized with PrimeScript™ RT Master Mix (Perfect Real Time) (Takara). The reactions were performed in accordance with the manufacturer’s instructions. The nucleotide sequences of the primers used to amplify cDNA fragments of *GAPDH*, *AK*, *UBC*, *ACT5C*, *ACT*, *RPS13*, *TUB*, *RPLP0* and *UCCR* for qPCR are shown in Table 2. PCR reactions were conducted in a S1000™ Thermal Cycler (Bio-Rad). The PCR products were then evaluated by 1.0% agarose gel electrophoresis. Bands of the expected sizes were excised and then each fragment was purified using a MiniBEST Agarose Gel DNA Extraction Kit Ver.3.0 (Takara).

**Table 2:** Primer pairs used for qPCR.

Gene name (Abbreviation)	Accession No	Sequence (5′-3′)	Product length (bp)	PCR efficiencies (%)	*R*^2^
Glyceraldehyde-3-phosphate dehydrogenase (*GAPDH*)	HQ012003	F: CTGATGCTCCCATGTTCGTG	202	99.4	0.9971
		R: CCAGAGGGTCCATCAACAGT			
Arginine kinase (*AK*)	HQ840714	F: GACCTTCTTGGTATGGTGCAATGAAG	175	109.4	0.9972
		R: GTTGGTAGGGCAGAAAGTGAGGA			
Ubiquitin C (*UBC*)	XM_022968576	F: CCTTGACGGGTAAAACTATTACGCTTGAA	206	109.5	0.9998
		R: GCCACGGAGACGCAGAACA			
Actin-5C (*ACT5C*)	XM_022975529	F: CGAGAAATCGTGCGTGACAT	244	94.0	0.9994
		R: CGTCGCACTTCATGATGGAG			
Actin (*ACT*)	XM_022981497	F: CACCTTCTACAACGAGCTGC	174	96.9	0.9984
		R: CCAGAGGCGTACAGAGAGAG			
Ribosomal protein S13 (*RPS13*)	XM_022972107	F: GTATGCACGCACCTGGTAAG	185	106.4	0.9977
		R: TCTGACTTGTGCGACTCCAT			
Tubulin (*TUB*)	XM_022962426	F: GACAACGAGGCCCTATACGA	205	91.2	0.9985
		R: CGAATCCGGGCATGAAGAAG			
Acidic ribosomal protein P0 (*RPLP0*)	XM_022968776	F: GGAAACCAACCCAGCTCTTG	198	102.2	0.9968
		R: GTCTTCTCAGGACCCAGACC			
Ubiquinol-cytochrome c reductase (*UCCR*)	XM_022974041	F: GGGCAATTCTCTTTTCATCTCACCCA	227	106.6	0.9997
		R: CACCCATTTCTTTCCTCAAATCTCCACC			

### Quantitative real-time PCR

qRT-PCR was performed using a qTOWER^3^ Real-Time PCR Thermal Cycler (Analytik Jena, Germany) with SYBR® Premix Ex TaqTM II (Tli RNaseH Plus) (Takara). The relative expression levels of mRNA were calculated with the ΔΔCT method^63^. The thermal cycling conditions were as follows: 95 °C for 3 min followed by 40 cycles of 95 °C for 15 s, 60 °C for 30 s and 72 °C for 30 s. Melting curve analysis from 65 to 95 °C was carried out after qPCR to ensure product specificity. The PCR amplification efficiency was analyzed by using different dilutions of the cDNA template. The standard curve for each gene was prepared according to the CT values at different cDNA concentrations. Primers with approximately 100% efficiency (*R*^2^ > 0.97) were used for qPCR (Table 2).

### Statistical analyses

The CT values from the qPCRsoft for qTOWER^3^ (Analytik Jena) were analyzed in Microsoft Excel. IBM® SPSS® Statistics version 23 (SPSS, Inc., Armonk, NY, USA) was used for one-way analysis of variance (ANOVA), Duncan’s multiple range test (DMRT) and linear regression analysis. The residuals of CT values were evaluated by the difference between the actual value and the value calculated from linear regression for each gene (Fig. 1). The stabilities of the nine candidate reference genes were evaluated using geNorm version 3.5 (http://medgen.ugent.be/genorm/), NormFinder_0953 (http://www.mdl.dk), BestKeeper, RefFinder (https://blooge.cn/RefFinder/) and the comparative delta CT method. The raw CT values were transformed into 2^(-delta CT)^ for geNorm and NormFinder analysis. geNorm calculated the average expression stability value (M) for each gene, and compared the pairwise variation V to determine the optimal number of control genes for normalization. NormFinder software compared the estimated inter- and intra-group variances to identify the best gene with the lowest stability value. BestKeeper software used raw data, and ranked the stability by standard deviation (SD) and coefficient of variation (CV). RefFinder software integrated geNorm, NormFinder, BestKeeper and the comparative delta CT method, to rank the candidate reference genes with the geomean of the ranking values.

### Supplementary Information

Supplementary Information.

## Data Availability

The data that support the findings of this study are available from Figshare, https://doi.org/10.6084/m9.figshare.24539974.

## References

[1] Qin HG, Wang DD, Ding J, Huang RH, Ye ZX (2006). Host plants of *Spodoptera litura*. Acta Agricult. Jiangxi.

[2] Shad SA (2012). Field evolved resistance to carbamates, organophosphates, pyrethroids, and new chemistry insecticides in *Spodoptera litura* Fab. (Lepidoptera: Noctuidae). J. Pest Sci..

[3] Sang S, Wang Z, Qi JW, Shu BS, Zhong GH (2013). Research progresses on pesticide resistance of *Spodoptera litura*. J. Environ. Entomol..

[4] Pan F (2014). Monitoring on the resistance of *Spodoptera litura* (Fabricius) to fifteen kinds of pesticides in Hainan region. Acta Agricult. Univ. Jiangxiensis.

[5] Su XN (2021). Control effects of biocontrol strain *Isaria javanica* on *Spodoptera litura* (Fabricius) and evaluation safety. J. South. Agricult..

[6] Zhang Z (2021). Identification and pathway analysis of genes related to binge eating in taro caterpillar *Spodoptera litura* 4th instar larvae. J. Plant Protect..

[7] Rao GV, Wightman JA, Rao DV (1993). World review of the natural enemies and diseases of *Spodoptera litura* (F.) (Lepidoptera: Noctuidae). Int. J. Trop. Insect Sci..

[8] Higuchi H, Yamamoto H, Suzuki Y (1994). Analysis of damage to soybeans infested by the common cutworm, *Spodoptera litura* Fabricius (Lepidoptera: Noctuidae). II. Estimation of leaf area damaged by young larvae using spectral reflectivity. Jpn. J. Appl. Entomol. Z..

[9] Patnaik, H. P. Pheromone trap catches of *Spodoptera litura* F. and extent of damage on hybrid tomato in Orissa. Advances in IPM for horticultural crops. Proceedings of the First National Symposium on Pest Management in Horticultural Crops: environmental implications and thrusts, Bangalore, India, 68–72 (1998).

[10] Maree JM, Kallar SA, Khuhro RD (1999). Relative abundance of *Spodoptera litura* F. and *Agrotis ypsilon* Rott. on cabbage. Pak. J. Zool..

[11] Matsumoto K (2000). Fast-growing leguminous trees in Indonesia and their insect pests. Trop. For..

[12] Qin H, Ye Z, Huang S, Ding J, Luo R (2004). The correlations of the different host plants with preference level, life duration and survival rate of *Spodoptera litura* Fabricius. Chin. J. Eco-Agriculture.

[13] Muthusamy R, Vishnupriya M, Shivakumar MS (2014). Biochemical mechanism of chlorantraniliprole resistance in *Spodoptera litura* (Fab) (Lepidoptera: Noctuidae). J. Asia-Pac. Entomol..

[14] Rehan A, Freed S (2014). Selection, mechanism, cross resistance and stability of spinosad resistance in *Spodoptera litura* (Fabricius) (Lepidoptera: Noctuidae). Crop Prot..

[15] Saleem M, Hussain D, Ghouse G, Abbas M, Fisher SW (2016). Monitoring of insecticide resistance in *Spodoptera litura* (Lepidoptera: Noctuidae) from four districts of Punjab, Pakistan to conventional and new chemistry insecticides. Crop Prot..

[16] Wang XG (2018). Insecticide resistance and enhanced cytochrome P450 monooxygenase activity in field populations of *Spodoptera litura* from Sichuan, China. Crop Prot..

[17] Xu L (2020). Transcriptome analysis of *Spodoptera litura* reveals the molecular mechanism to pyrethroids resistance. Pestic Biochem. Physiol..

[18] Kaur T, Vasudev A, Sohal SK, Manhas RK (2014). Insecticidal and growth inhibitory potential of Streptomyces hydrogenans DH16 on major pest of India, *Spodoptera litura* (Fab.) (Lepidoptera: Noctuidae). BMC Microbiol..

[19] Bi H (2022). CRISPR/Cas9-mediated *Serine protease 2* disruption induces male sterility in *Spodoptera litura*. Front. Physiol..

[20] Zimmermann G (2007). Review on safety of the entomopathogenic fungus *Metarhizium anisopliae*. Biocontrol Sci. Techn..

[21] Pathak, D., Yadav, R. & Kumar, M. Microbial pesticides: development, prospects and popularization in India in *Plant-Microbe Interactions in Agro-Ecological Perspectives* (eds. Singh, D. P. *et al.*) 455–471 (Springer, 2017).

[22] Han JH, Jin BR, Kim JJ, Lee SY (2014). Virulence of entomopathogenic fungi *Metarhizium anisopliae* and *Paecilomyces fumosoroseus* for the microbial control of *Spodoptera exigua*. Mycobiology.

[23] Hong M, Peng G, Keyhani NO, Xia Y (2017). Application of the entomogenous fungus, *Metarhizium anisopliae*, for leafroller (*Cnaphalocrocis medinalis*) control and its effect on rice phyllosphere microbial diversity. Appl. Microbiol. Biot..

[24] Wang YS (2018). Efficacy of *Empedobacter brevis* on controlling *Mythimna separate* (Walker) in corn field. Agrochemicals.

[25] Hu X (2020). Development and application of efficient *Bacillus thuringiensis* KN11 insecticides. Chin. J. Biol. Control.

[26] Hu F (2021). Control efficacy of biopesticide *Bacillus thuringiensis* G033A combined with reduced low dose chemical pesticides on *Spodoptera frugiperda*. Chin. J. Biol. Control.

[27] Hu F (2023). Control efficacy of *Bacillus thuringiensis* tiny microgranules on maize Lepidopteran pests. Chin. J. Biol. Control.

[28] Perumal V (2023). First report on the enzymatic and immune response of *Metarhizium majus* bag formulated conidia against *Spodoptera frugiperda*: An ecofriendly microbial insecticide. Front. Microbiol..

[29] Ferré, J., Van Rie, J. & MacIntosh, S. C. Insecticidal genetically modified crops and insect resistance management (IRM) in *Integration of Insect-resistant Genetically Modified Crops within IPM Programs* (eds. Romeis, J., Shelton, A. M. & Kennedy, G. G.) 41–85 (Springer Science and Business Media, 2008).

[30] Sumerford DV, Head GP, Shelton A, Greenplate J, Moar W (2013). Field-evolved resistance: Assessing the problem and ways to move forward. J. Econ. Entomol..

[31] Tabashnik BE, Brevault T, Carriere Y (2013). Insect resistance to Bt crops: lessons from the first billion acres. Nat. Biotechnol..

[32] Park Y (2014). ABCC transporters mediate insect resistance to multiple Bt toxins revealed by bulk segregant analysis. BMC Biol..

[33] Yao X (2022). ABCC2 is a functional receptor of *Bacillus thuringiensis* Cry1Ca in *Spodoptera litura*. Int. J. Biol. Macromol..

[34] Chen H, Yin Y, Li Y, Mahmud MS, Wang Z (2012). Identification and analysis of genes differentially expressed in the *Spodoptera litura* fat body in response to the biocontrol fungus, *Nomuraea rileyi*. Comp. Biochem. Physiol. B Biochem. Mol. Biol..

[35] Lu Y (2013). Identification and validation of reference genes for gene expression analysis using quantitative PCR in *Spodoptera litura* (Lepidoptera: Noctuidae). PLoS One.

[36] Lu Y (2020). Comparative genomic analysis of C-type lectin-domain genes in seven holometabolous insect species. Insect Biochem. Mol. Biol..

[37] Xia X, You M, Rao XJ, Yu XQ (2018). Insect C-type lectins in innate immunity. Dev. Comp. Immunol..

[38] Zhu Y, Yu X, Cheng G (2020). Insect C-Type lectins in microbial infections. Adv. Exp. Med. Biol..

[39] Scharlaken B (2008). Reference gene selection for insect expression studies using quantitative real-time PCR: The head of the honeybee, *Apis mellifera*, after a bacterial challenge. J. Insect Sci..

[40] Lord JC, Hartzer K, Toutges M, Oppert B (2010). Evaluation of quantitative PCR reference genes for gene expression studies in *Tribolium castaneum* after fungal challenge. J. Microbiol. Methods.

[41] Shu Y, Du Y, Wang J (2011). Molecular characterization and expression patterns of *Spodoptera litura* heat shock protein 70/90, and their response to zinc stress. Comp. Biochem. Physiol. B.

[42] Shu BS (2018). Evaluation of reference genes for real-time quantitative PCR analysis in larvae of *Spodoptera litura* exposed to azadirachtin stress conditions. Front. Physiol..

[43] Lland, H., Hertzberg, M. & Marlton, P. Myeloid leukenmia in *Methods and Protocols* (ed. Colgan, S. P.) 53 (Humana, 2006).

[44] Karlen Y, McNair A, Perseguers S, Mazza C, Mermod N (2007). Statistical significance of quantitative PCR. BMC Bioinform..

[45] Burns M, Valdivia H (2008). Modelling the limit of detection in real-time quantitative PCR. Eur. Food Res. Technol..

[46] Bustin SA (2009). The MIQE guidelines: minimum information for publication of quantitative real-time PCR experiments. Clin. Chem..

[47] Wan H (2010). Selection of appropriate reference genes for gene expression studies by quantitative real-time polymerase chain reaction in cucumber. Anal. Biochem..

[48] Möller, W. & Maassen, J. A. On the structure, function and dynamics of L7/L12 from *Escherichia coli* ribosomes in *Structure Function and Genetics of Ribosomes* (eds. Hardesty, B. & Kramer, G.) 309–325 (Springer-Verlag, 1986).

[49] Hasler P, Brot N, Weissbach H, Parnassa AP, Elkon KB (1991). Ribosomal proteins P0, P1, and P2 are phosphorylated by casein kinase II at their conserved carboxyl termini. J. Biol. Chem..

[50] Rodriguez-Gabriel MA, Remacha M, Ballesta JP (1998). Phosphorylation of ribosomal protein P0 is not essential for ribosome function but can affect translation. Biochemistry.

[51] Yacoub A, Kelley MR, Deutsch WA (1996). *Drosophila* ribosomal protein P0 contains apurinic/apyrimidinic endonuclease activity. Nucleic. Acids Res..

[52] Frolov MV, Birchler JA (1998). Mutation in P0, a dual function ribosomal protein/apurinic/apyrimidinic endonuclease, modifies gene expression and position effect variegation in *Drosophila*. Genetics.

[53] Craig TL, Denlinger DL (2000). Sequence and transcription patterns of 60S ribosomal protein P0, a diapause-regulated AP endonuclease in the flesh fly, *Sarcophaga crassipalpis*. Gene.

[54] Cukras AR, Southworth DR, Brunelle JL, Culver GM, Green R (2003). Ribosomal proteins S12 and S13 function as control elements for translocation of the mRNA:tRNA complex. Mol. Cell.

[55] Van Hiel MB (2009). Identification and validation of housekeeping genes in brains of the desert locust *Schistocerca gregaria* under different developmental conditions. BMC Mol. Biol..

[56] Sun W, Jin Y, He L, Lu WC, Li M (2010). Suitable reference gene selection for the different strains and developmental stages of the carmine spider mite, *Tetranychus cinnabarinus*, using quantitative real-time PCR. J. Insect Sci..

[57] Majerowicz D (2011). Looking for reference genes for real-time quantitative PCR experiments in *Rhodnius prolixus* (Hemiptera: Reduviidae). Insect Mol. Biol..

[58] Mamidala P, Rajarapu SP, Jones SC, Mittapalli O (2011). Identification and validation of reference genes for quantitative real-time polymerse chain reaction in *Cimex lectularius*. J. Med. Entomol..

[59] Koramutla MK, Aminedi R, Bhattacharya R (2016). Comprehensive evaluation of candidate reference genes for qRT-PCR studies of gene expression in mustard aphid, *Lipaphis erysimi* (Kalt). Sci. Rep-UK.

[60] Ibanez F, Tamborindeguy C (2016). Selection of reference genes for expression analysis in the potato psyllid, *Bactericera cockerelli*. Insect Mol. Biol..

[61] Dzaki N, Ramli KN, Azlan A, Ishak IH, Azzam G (2017). Evaluation of reference genes at different developmental stages for quantitative real-time PCR in *Aedes aegypti*. Sci. Rep-UK.

[62] Chen QJ, Li GH, Pang Y (2000). A simple artificial diet for mass rearing of some noctuid species. Entomol. Knowl..

[63] Livak KJ, Schmittgen TD (2001). Analysis of relative gene expression data using real-time quantitative PCR and the 2^-ΔΔCT^ method. Methods.

