# Supplementary material for: Determination of internal controls for quantitative gene expression of Spodoptera litura under microbial pesticide stress
Source: Sci Rep. 2024 Mar 13;14:6143. doi: 10.1038/s41598-024-56724-9 (PMC10937984; doi:10.1038/s41598-024-56724-9)
Supplement: Supplementary file 1 — Supplementary Information. [file 41598_2024_56724_MOESM1_ESM.pdf]

Supplementary Information

Determination of internal controls for quantitative gene expression of *Spodoptera litura* under microbial pesticide stress

Shuang Wu[1], Yunmi Luo[1], Zhihong Zeng[1], Ying Yu[1], Shicai Zhang[1], Yan Hu[1], Lei Chen*[1]

[1]Institute of Vegetable and Flower Research, Chongqing Academy of Agricultural Sciences, Chongqing 401329, China

*Corresponding author. email: chenlei112301@163.com

Supplementary Table S1. The total RNA concentrations and A260/A280 ratios determined with GeneQuant Pro RNA/DNA Calculator for the RNA samples.

Treatments		Time after treatment	Average concentration (ng·µL^{-1})	A260/A280 ratios
direct treatment conditions	Control	6 h	1057.3	2.075
		12 h	1196.0	2.091
		24 h	1036.6	2.079
		48 h	779.4	2.085
		72 h	1397.4	2.079
	M. anisopliae-infected	6 h	1015.8	2.087
		12 h	791.3	2.093
		24 h	963.5	2.072
		48 h	1145.8	2.099
		72 h	921.1	2.080
	E. brevis-infected	6 h	964.1	2.107
		12 h	711.3	2.043
		24 h	1236.7	2.068
		48 h	736.9	2.066
		72 h	1169.0	2.086
	B. thuringiensis-infected	6 h	1136.6	2.035
		12 h	1654.8	2.106
		24 h	1169.1	2.088
		48 h	915.3	2.080
		72 h	1014.7	2.103
indirect treatment conditions	Control	24 h	1089.7	1.973
		48 h	1228.1	1.914
		72 h	1158.5	2.077
	M. anisopliae-infected	24 h	1199.1	1.974
		48 h	1658.2	2.072
		72 h	1333.8	2.055
	E. brevis-infected	24 h	950.4	1.937
		48 h	1256.7	2.088
		72 h	810.9	2.109
	B. thuringiensis-infected	24 h	893.7	1.995
		48 h	971.3	1.953
		72 h	1674.3	2.090
comprehensive treatment conditions	Control	24 h	994.5	2.069
		48 h	767.9	2.065
		72 h	920.0	2.074
	M. anisopliae-infected	24 h	835.8	2.068
		48 h	1183.9	2.061
		72 h	905.3	2.054
	E. brevis-infected	24 h	864.7	2.047
		48 h	877.6	1.966
		72 h	991.9	2.086
	B. thuringiensis-infected	24 h	1284.1	1.926
		48 h	1249.7	1.957
		72 h	849.6	2.116

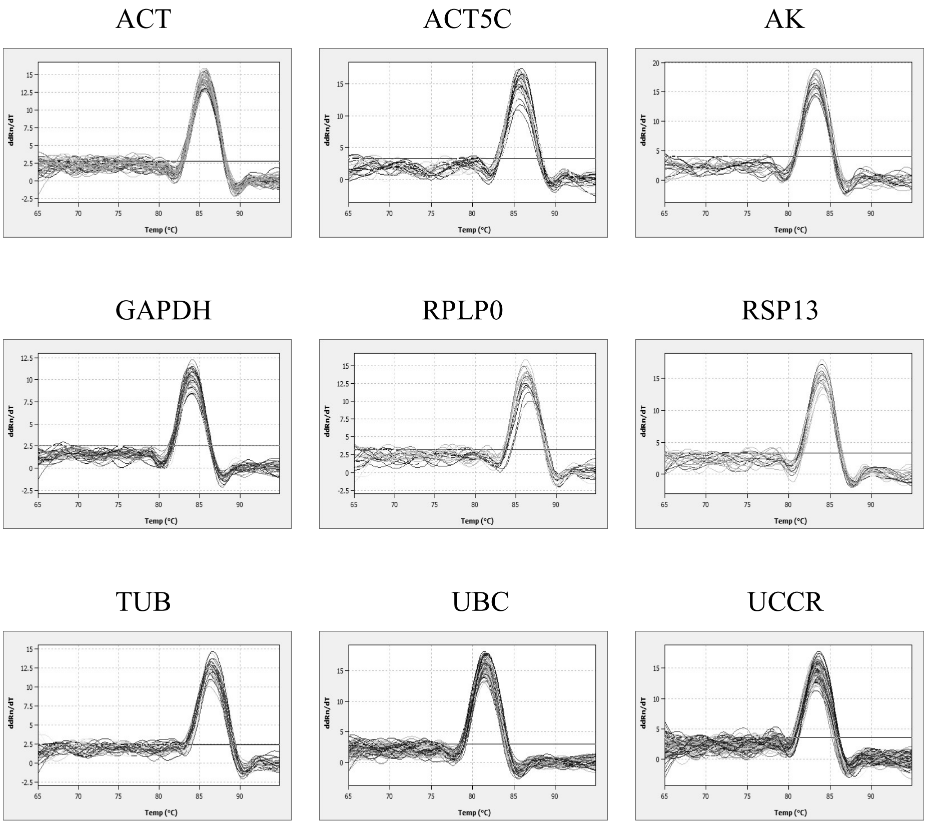

Supplementary Figure S1. Melting curve analyses of PCR products for nine candidate reference genes.

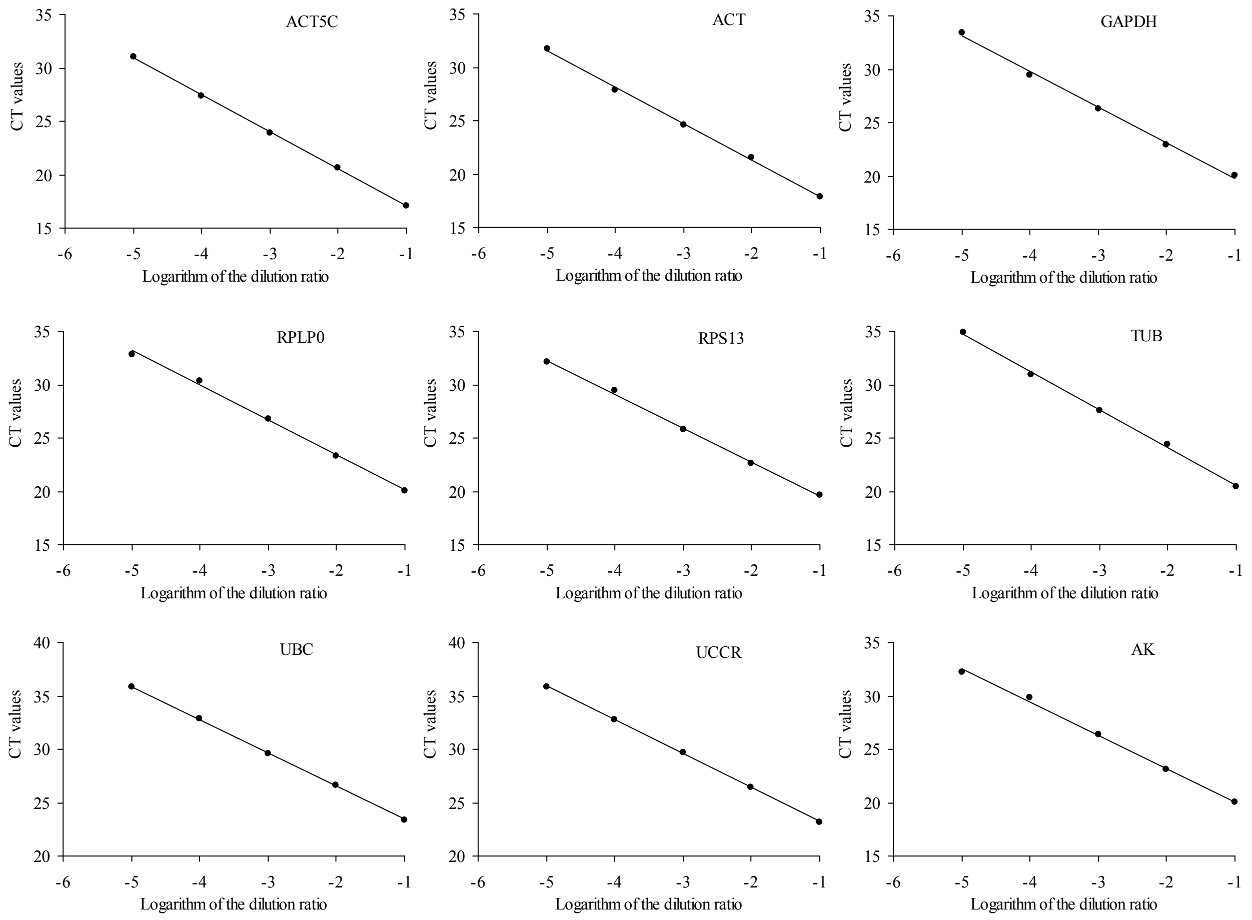

Supplementary Figure S2. Dilution curves for determination of the PCR efficiencies. The average CT value for three technical replicates for each concentration was marked as solid circle.
